# Piloting and evaluating feasibility of a training program to improve patient safety for inter-professional inpatient care teams – study protocol of a cluster randomized controlled trial

**DOI:** 10.1186/s13063-019-3448-7

**Published:** 2019-06-28

**Authors:** Julia Dinius, Antje Hammer, Tanja Manser, Corinna Bergelt, Levente Kriston, Mirjam Körner

**Affiliations:** 1 Medical Psychology and Medical Sociology, Medical Faculty, Albert-Ludwigs-University, Freiburg, Germany; 2 Institute for Patient Safety, University Hospital Bonn, Bonn, Germany; 3 FHNW School of Applied Psychology, University of Applied Sciences and Arts Northwestern Switzerland, Olten, Switzerland; 4 Department of Medical Psychology, University Medical Center Hamburg-Eppendorf, Hamburg, Germany

**Keywords:** Patient safety, Inpatient care, Inter-professional teamwork, Patient involvement, Error management, E-learning, In-person training, Cluster randomized controlled trial, Evaluation

## Abstract

**Background:**

Improving patient safety is a major goal in healthcare systems worldwide. There are several international training programs to improve patient safety, but they are often focused on single topics and professions. Therefore, one inter-professional training program for inpatient care teams, which combines key areas of patient safety (Teamwork, Error management and Patient involvement), was developed by our research group. In the present study we aim to (1) pilot this training program by comparing two different training formats (e-learning only versus blended learning) with a waiting control group and (2) evaluate the feasibility of the intervention.

**Methods and analysis:**

(1) To pilot the intervention a cluster randomized controlled trial will be performed at three study sites. Therefore, an e-learning group and a blended learning group will be compared to a waiting control group at three points of assessment; (2) The feasibility of the intervention will be evaluated using qualitative methods. We will conduct problem-focused individual interviews as part of the post-intervention measurement in order to collect information on acceptance, implementation, promoting factors and barriers from the staffs’ perspective.

**Discussion:**

The study puts forth a training program which has the potential to improve patient safety in inpatient care. Members of inter-professional inpatient care teams can receive systematic training in three competencies which are central to patient safety management. Thus, we expect the greatest improvement in staff Safety-related behavior regarding Teamwork, Error management and Patient involvement as well as Subjectively perceived patient safety in the blended learning group. In addition, the development of an optimal implementation strategy can foster implementation of the intervention in healthcare practice. Consequently, the intervention could be used continuously and comprehensively for advanced training of hospital staff.

**Trial registration:**

The study has been registered in the German Register of Clinical Trials (DRKS-ID: DRKS00012818). Registered on August 8, 2017.

**Electronic supplementary material:**

The online version of this article (10.1186/s13063-019-3448-7) contains supplementary material, which is available to authorized users.

## Background

Improving patient safety is a major goal in healthcare systems worldwide [1]. Patient safety can be defined as the “avoidance, prevention and amelioration of adverse outcomes or injuries stemming from the process of healthcare” [2]. The World Health Organization has stated that 134 million adverse events occur each year in hospitals, contributing to 2.6 million deaths annually due to unsafe care [3]. Internationally, a wide range of interventions for improving patient safety exist, putting a particular emphasis on developing training programs [4]. Existing international training programs often focus on single professions as well as a specific patient safety topic, e.g., teamwork (Strategies and Tools to Enhance Performance and Patient Safety – TeamSTEPPS, USA) [5, 6], Speak up initiatives (USA) [7–9], patient involvement (Patientenempfehlungen (Recommendations for patients) – PATEM, Switzerland) [10]. To the best of our knowledge, there is no inter-professional training program for inpatient care teams which covers different key areas of patient safety. However, due to the complexity of patient safety, a reduction to the most important contents was necessary. Therefore, focus groups were established by our research group to find out the requirements of an inter-professional, multi-content, training program in Germany [11]. The main findings of the study were the training format and recommendations regarding the content: The training should be a combination of e-learning and interactive in-person training (blended learning), with the content focused on *Teamwork*, *Error management* and *Patient involvement* [11]. These recommendations are consistent with the Patient Safety Curriculum Guide [1] and the Learning Objective Catalog for Patient Safety [12], which define these as the three key areas, among others. If we look into the literature, a considerable amount of evidence can be found confirming these topics as the three key areas. Current research shows that training programs can provide new insights and improve attitudes toward teamwork [13–17]. Improving teamwork can lead to increased patient satisfaction [18], employee satisfaction [19] and employee well-being [20], more efficient patient treatment [21] as well as to decreased medical errors [22] and mortality [23]. By improving communication within the team of healthcare professionals and with patients, such programs can also contribute to improvements in healthcare delivery and make healthcare safer [17, 24–31]. Furthermore, discussions and reflections of adverse events, errors and their consequences for patients can build the foundation for open communication and thus a practiced safety culture [32]. With regard to patient involvement, patients have been found to possess the willingness, ability and desire to play an active role in error prevention in accordance with their capabilities [28, 33–36]. However, this willingness is closely linked to positive attitudes of the inpatient care team concerning open communication about errors and patient involvement as well as to their social and communicative competencies [37–39]. In total, all three topics are mutually dependent on each other and lead to better patient safety.

The KOMPAS project (KOMPAS = German acronym for “Development and evaluation of a complex training program to improve patient safety”) takes a first step to close this gap by designing a training program according to the stated criteria by using innovative adult education methods. Moreover, an optimal implementation strategy can foster the implementation of the intervention in healthcare practice by overcoming potential barriers. The present study aims to (1) pilot the training program by comparing two different training formats with a waiting control group (WCG) and (2) evaluate the feasibility of this intervention.

### Research questions

1. In the course of piloting the intervention, the following research question and hypothesis will be pursued:
  To what extent can the intervention be used to improve Safety-related behavior with regard to Teamwork, Error management and Patient involvement?
    H1: The improvement of Safety-related behavior with regard to Teamwork, Error management and Patient involvement in IG1 (intervention group 1: e-learning only) and IG2 (intervention group 2: combined e-learning and in-person training = blended learning) is significantly higher than in the WCG.
      In the e-learning course, the participants will work individually on the theoretical foundations for the three key areas. Due to the additional in-person training, the participants will try out and consolidate their knowledge. This leads us to the following hypothesis:
    H2: The greatest improvement in Safety-related behavior with regard to Teamwork, Error management and Patient involvement is in IG2 (intervention group 2: combined e-learning and in-person training = blended learning)
  To what extent can the intervention be used to improve Subjectively perceived patient safety?
    H3: The improvement of Subjectively perceived patient safety in IG1 (intervention group 1: e-learning only) and IG2 (intervention group 2: combined e-learning and in-person training = blended learning) is significantly higher than in the WCG
    H4: The greatest improvement of Subjectively perceived assessed patient safety is in IG2 (intervention group 2: combined e-learning and in-person training = blended learning)
2. With regard to evaluating the feasibility of the intervention the following research question should be answered:
  Which facilitators and barriers for successful implementation of the intervention can be identified?

## Methods and analysis

### Study design

According to the Medical Research Council Framework for Developing and Evaluating Complex Interventions [40], the study is classified as Phase 2 (Feasibility and Piloting).

**Fig. 1:**
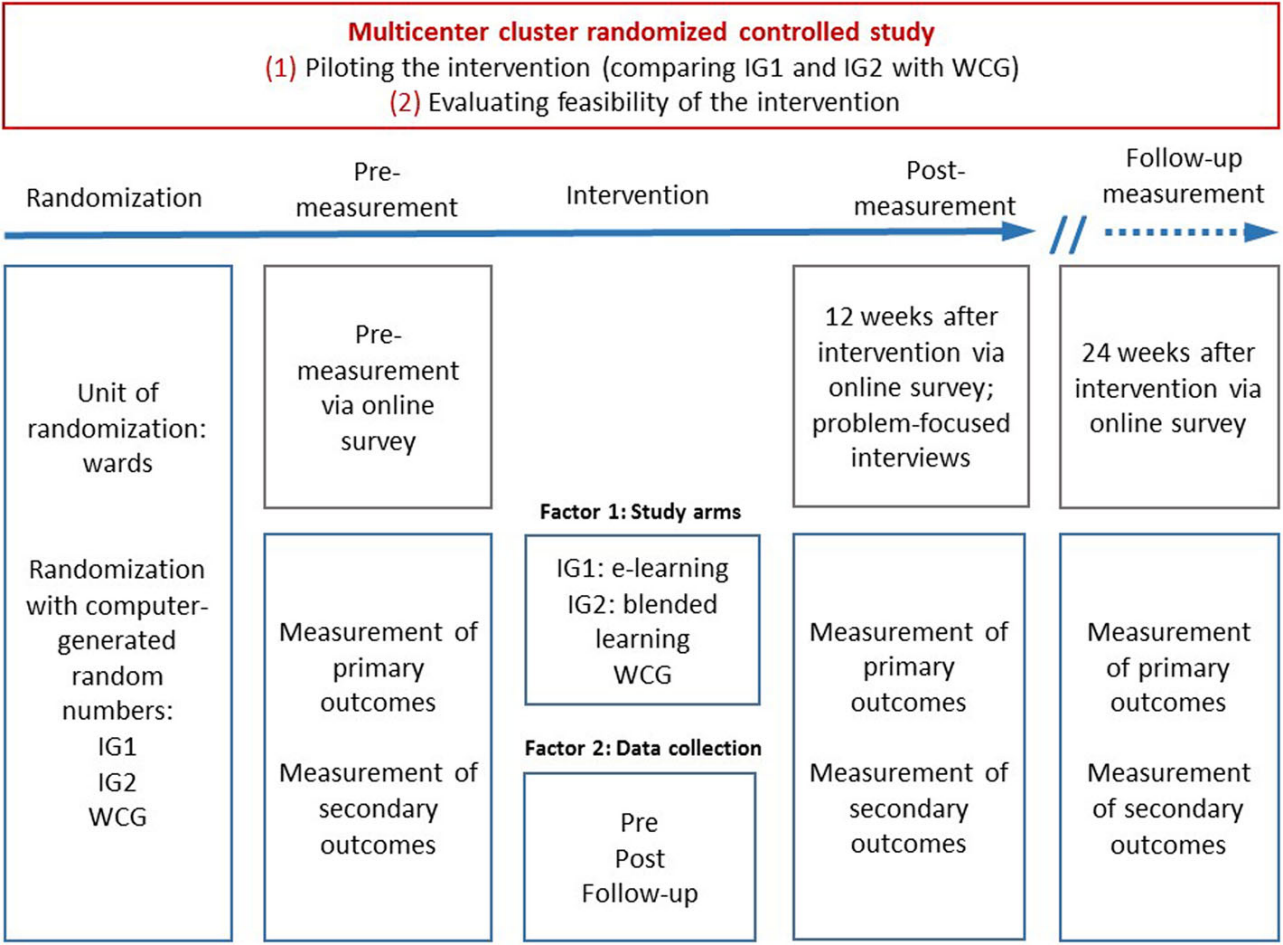
Study process. *IG1* intervention group 1, *IG2* intervention group 2, *WCG* waiting control group

To pilot the intervention, a cluster randomized controlled study will be performed. Cluster units will be wards at the participating hospitals. The cluster design was selected as it is considered the best option if an individual randomization is not possible. For methodical, ethical and organizational reasons, the same intervention must be offered to all staff on a ward, otherwise there is a risk of contamination [41]. Prior to data collection, inpatient care teams from the participating wards (clusters) at the three study sites will randomly be assigned the to: (1) an e-learning group (intervention group 1; IG1), (2) a blended learning group (combined e-learning and in-person training; intervention group 2; IG2), or (3), a WCG based on computer-generated randomization sequence with a 1:1:1 treatment allocation ratio, stratified by center. To ensure concealment, central randomization with variable block sizes will be conducted by an independent statistician not involved in recruitment or implementation of the intervention. Both representatives of the clusters and work councils sought consent before randomization. Data will be collected at three points of assessment: (1) prior to the intervention (pre-measurement), (2) 12 weeks post intervention (post-measurement), and 24 weeks post intervention (follow-up measurement). Figure 1 illustrates the study process.

The study design will also be tested as part of the piloting process. This will involve reviewing the central components of the study, e.g., randomization, recruitment procedures, inclusion and exclusion criteria, comparability of samples, and participation rates. Likewise, the intervention effect and the intra-cluster correlation coefficient (ICC) will be estimated in order to provide a suitable basis for calculating the sample sizes for a future confirmatory study.

The feasibility of the intervention will be evaluated using qualitative methods. In the course of the post-measurements, problem-focused individual interviews (half standardized) will be conducted with a random selection of participants (for approximately 10% of the participants) from IG1 (*N* = 18) and IG2 (*N* = 18).

### Study setting and recruitment

Hospitals geographically located in areas around the three study sites (Hamburg (North-West Germany), Bonn (Middle-West Germany) and Freiburg (South-West Germany)) can participate in the study. A list of study sites can be obtained from consortium leaders in Freiburg. Hospital recruitment will be organized non-randomly by each of the three study sites according to pre-defined criteria for participating wards: (1) inpatient care teams with a minimum of 10 members, (2) inpatient care teams with an inter-professional composition. Pediatric, emergency and intensive care wards will be excluded from participating in the study, as teamwork is highly regimented in pediatrics and emergency care, and patient involvement would not be comparable to other wards. Moreover, inpatient care teams with less than 10 members and mono-professional teams will be excluded. Participating hospitals will be asked to assign a local study coordinator, who supports the research team in local staff recruitment, organization of the intervention, and data collection at the wards. The following inclusion criteria for the participants are mandatory: (1) being a member of the participating inter-professional inpatient care team (e.g., physicians, nurses, physiotherapists, speech therapists, ergo therapists), (2) being at least 18 years old and (3) being fluent in German. Team members of mono-professional teams, team members under the age of 18 years and team members who are not able to speak German fluently will be excluded from participating in the study.

### Sample size

As the anticipated effect of the intervention on the primary and secondary outcomes is unclear, one of the aims of the feasibility study is to estimate this effect, in preparation for a confirmatory study [40]. Thus, the required sample size is determined on the basis of the identification of important rare (predicted or unpredicted) events [42]. In order to identify an event which occurs in 10% of cases with a confidence interval of 95%, 30 people have to be investigated in each relevant subgroup [42]. A conservative minimum of 10 employees per inpatient care team on the wards (cluster) will be taken as a starting point, so the three study sites each comprise nine wards (three per study arm). This means that viable data from a total of 90 participants (30 in IG1, IG2 and WCG, respectively) must be collected at each study site. As we anticipate a dropout rate of up to 25%, 12 wards will be recruited at each study site and randomly assigned to the three study arms. Thus, the study will in total include 36 wards with a minimum of 360 participants across three sites (10 participants * 4 wards * 3 study arms * 3 study sites).

### Intervention

The intervention is didactically based on adult learning theories [43] and consists of a combination of e-learning and inter-professional, behavior-based team training performed in-person (blended learning) and involving a high level of interaction among team members. With regard to the content, basic competencies for patient safety will be imparted in the three key areas *Teamwork*, *Error management,* and *Patient involvement*.

In the following, the intervention is described according to the Templates for Intervention Description and Replication (TIDieR) [44].

**Fig. 2:**
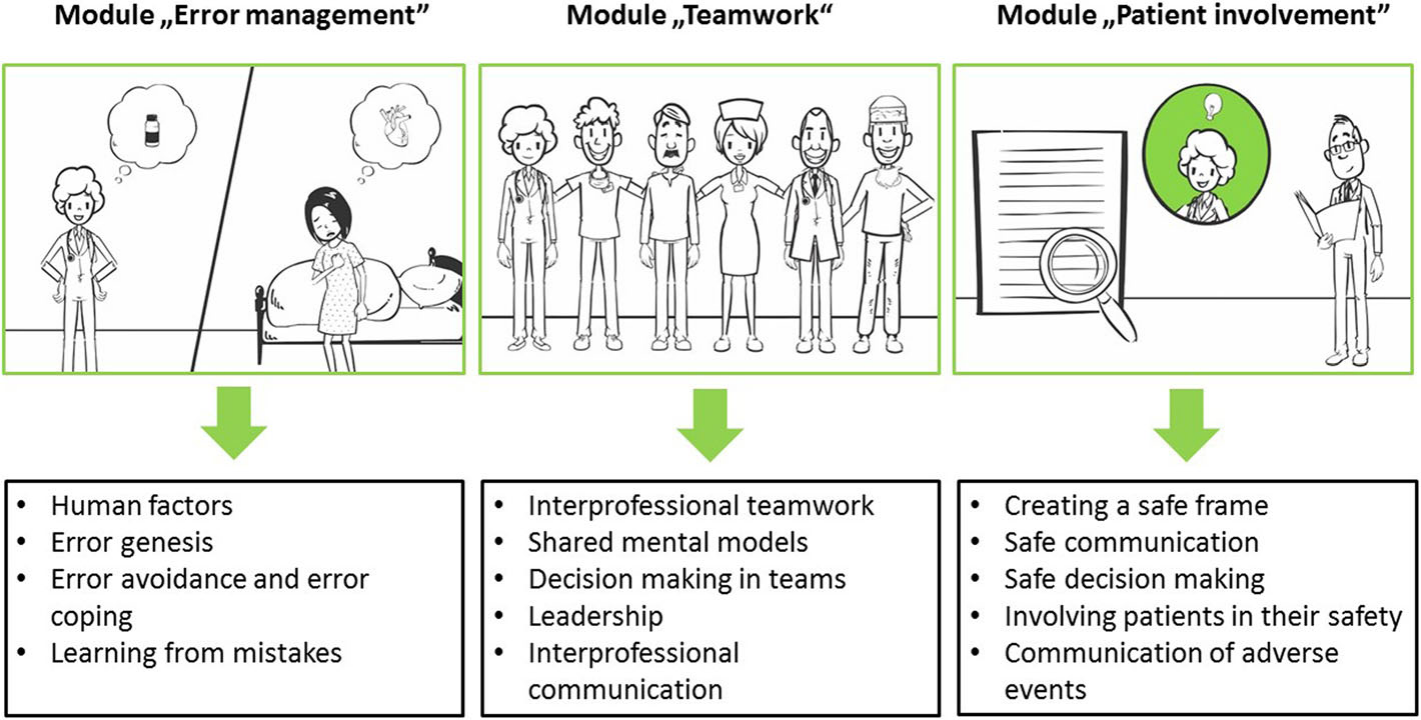
Overview over the submodules

Participants can access the online e-learning platform via a link. The e-learning course can be completed individually independent of time and location and allows for self-directed learning [45]. First, a video is presented, which introduces the topic of patient safety and demonstrates how to handle the e-learning course. For each key area a 1-h module is provided. The three modules are divided into submodules, which last from 5 to 10 min. The participants can go through as many single submodules, as they have time in a row. They have the opportunity to interrupt the process and restart from the same point at a later time. Within each module, an introduction video, presenting an overview of the content of the module, is provided. A variety of learning materials are used (instructional videos, interactions within text-based and audio-visual content). A section with take-home information concludes each module. Figure 2 provides an overview over the submodules. As an incentive, physicians get Continuing Medical Education (CME) credits and registered nurses get continuing education credits.

The participants have a maximum of 6 weeks to complete the whole e-learning course. They will be reminded twice (after 2 weeks and after 4 weeks). Moreover, a poster in their ward rooms reminds them to participate. In addition, they will receive cups and pens with the study logo as encouragement to participate.

The in-person training will take place in the actual teams in which the participants work. It will be conducted 1 to 4 weeks after the participants have completed the e-learning course. Behavior-related, practical exercises corresponding to the three key areas will be performed to facilitate the transfer of the theoretical content from e-learning into routine practice. Within the key area *Teamwork*, a video analysis will be conducted. Therefore, participants have to find mistakes in inter-professional teamwork seen in the video (corresponding to the e-learning submodules) and make appropriate recommendations for improving the inter-professional teamwork. Within the key area *Error management*, they have to do a detailed case analysis of a provided CIRS (Critical Incident Reporting System) case with the help of an Ishikawa chart [46]. Within the key area *Patient involvement*, the participants have to act out a role play on the subject “Communicate an adverse event.” Participants’ experiences and challenges regarding communication and behavior will be discussed after the role play. At the end, an action plan including important take-home information for each key area will be established.

The in-person training is expected to last for 210 min including breaks, which is considered realistic and resource-friendly. It will be conducted by psychologists, educationalists, nurses and public health scientists of our research team with experience in group moderation and adult education courses. These moderators will be trained in a train-the-trainer workshop by the consortium leaders. A trainer manual will provide the basis for the training and will contain information on the framework conditions (location, premises, duration and participants), the training schedule, and the content of the three key areas (including objectives, methods, and materials).

**Fig. 3:**
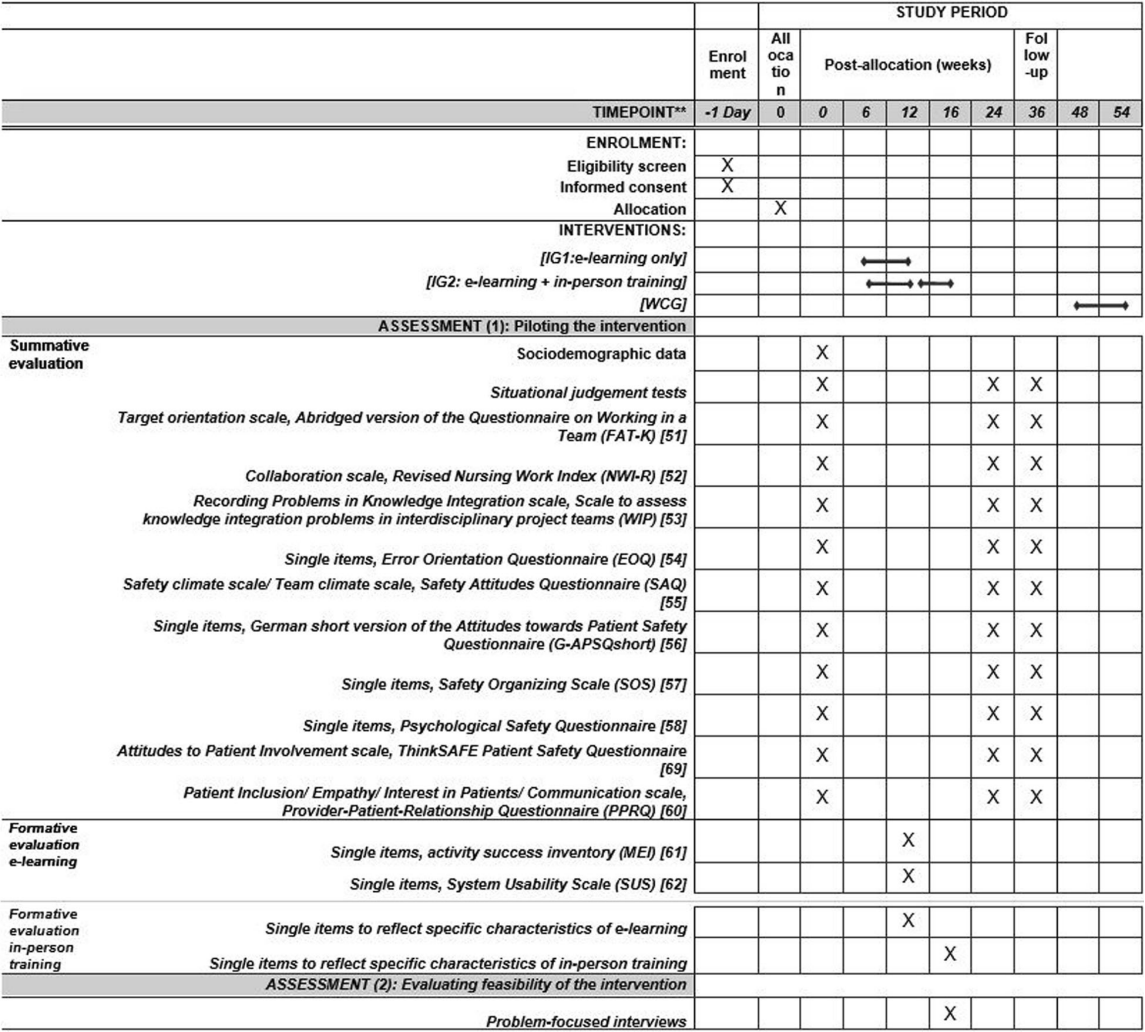
Schedule of enrolment, interventions and assessments (as per Standard Protocol Items: Recommendations for Interventional Trials (SPIRIT) Figure [63])

The intervention promotes all components of the KSA framework (Knowledge, Skills, Abilities) [47] in the three key areas. The intervention will take place at an employee level (e-learning) and a team level (inter-professional in-person training), but will also address the organizational and patient level. The intervention will be carried out at cluster level. In accordance with a flipped classroom approach (the trainees will prepare for the in-person training through e-learning), the participants of IG2 (blended learning group) will start with the e-learning course, where they work individually on the theoretical foundations for the three key areas (knowledge). Subsequently, the participants of IG2 will try out (skills) and consolidate (abilities) their knowledge in the in-person training. IG1 participants will work exclusively on the e-learning course. The WCG will not initially be assigned to an intervention, but will be given the opportunity to participate once the follow-up measurement has been concluded (see Fig. 3). Figure 3 shows a schedule of study enrolment, interventions and assessments. The recommendations for interventional trials (SPIRIT) checklist [63] is available as Additional file 1.

### Outcomes

The primary outcomes in the pilot study are *Safety-related behavior regarding Teamwork, Error management and Patient involvement* and S*ubjectively perceived patient safety.* The secondary outcomes are aspects of *Teamwork*, *Error management* and *Patient involvement*.

### Measuring the outcomes

As part of piloting the intervention, all outcomes will be measured at the individual level. Primary and secondary outcomes will be assessed at three points in time (pre, post and follow-up) by an online survey for the purpose of a summative evaluation. It will take approximately 20 min to complete this survey. There is also a paper version of the survey available. For the primary outcomes, situational judgement tests [48–50] will be used. They were pretested within an expert workshop with different members of inter-professional teams (e.g., physicians, nurses, therapists). The situational judgement tests describe typical everyday situations specifying five possible realistic behaviors. The participants have to rank the behaviors in the way that they most likely would react (ranging from (1) this approach is closest to my reaction, to (5) this approach is the least of my reactions). The ideal sequence, based on results of the expert workshop, will be scored with 30 points (4*4 + 3*3 + 2*2 + 1*1 + 0*0). The worst sequence will be scored with 10 points (4*0 + 3*1 + 2*2 + 1*3 + 0*4). The secondary outcomes will be assessed by validated scales and single items from standardized questionnaires. (e.g., *Teamwork* = Abridged version of the Questionnaire on Working in a Team (FAT-K) [51], Revised Nursing Work Index (NWI-R) [52], Scale to assess knowledge integration problems in interdisciplinary project teams (WIP) [53]; *Error management* = Error Orientation Questionnaire (EOQ) [54], Safety Attitudes Questionnaire (SAQ) [55], German short version of the Attitudes towards Patient Safety Questionnaire (G-APSQ short) [56] Safety Organizing Scale (SOS) [57], Psychological Safety Questionnaire [58]; *Patient involvement:* ThinkSAFE Patient Safety Questionnaire [59], Provider-Patient-Relationship Questionnaire (PPRQ) [60]. Items will be answered on 5-point Likert scales (ranging from totally disagree (1) to totally agree (5). A list of all included questionnaires is provided in Fig. 3.

Once the intervention components (e-learning, in-person training) have been completed, the feasibility of the intervention will be investigated for the purposes of a formative evaluation. To evaluate the e-learning, a short online survey will be conducted with all e-learning participants. To evaluate the in-person training, a paper-pencil survey will be conducted with all in-person training participants. Both surveys will last approximately 5 min. The intervention components will be evaluated with regard to satisfaction and acceptance. Additionally, the user-friendliness of the e-learning course will be measured. Therefore, validated scales from standardized questionnaires will be used [61, 62]. Furthermore, items will be designed which reflect the specific characteristics of e-learning (e.g., the option of interrupting the e-learning course and restarting from the same point).

To evaluate the feasibility of the intervention, problem-focused individual interviews will be conducted. Within these interviews, the acceptance, feasibility and user-friendliness of the intervention components will be examined from the perspective of the participants, and barriers to participation and promoting factors, which could have an impact on primary and secondary outcomes, will be explored.

Figure 3 provides the schedule of enrollment, interventions and assessments.

### Data collection and management

To access the online survey regarding summative evaluation, all participants will receive an email with a unique link to the online survey at each time of assessment (pre, post and follow-up) (see Fig. 1) from the local study coordinator. Lists with contact details of participants will be stored by the local study coordinator at participating hospitals. None of the researchers at the study sites will have access to any contact information of the participants. If required, participants can download a paper version of the survey and return questionnaires by mail to the study sites. Moreover, wards that do not provide email access for participants will receive a paper version of the survey coded with the ward number for unambiguous allocation of returned surveys. With the latter option, questionnaires will be collected in a survey mailbox at the wards or can be mailed directly to the study sites. For ensuring the allocation of individual paper-pencil and online data for pre, post and follow-up surveys, participants will be asked to create an individual four-digit ID to be entered in the survey. In order to raise response rates at each time of assessment, participants will receive at least two reminders via email 2 and 4 weeks after the initial invitation to the survey. In case of distributed paper-pencil questionnaires, the local study coordinator will send at least two postal reminders or give verbal reminders in team meetings at the wards. In addition, a poster in the ward rooms will remind them to participate.

Online surveys (pre, post and follow-up) will be entered by participants and pseudonyms stored on the survey projects at QuestBack server park (located in Bremen, Germany), a BSI-certified data center ensuring stringent security requirements. Paper-based questionnaires collected at the three study sites will be entered to the online survey platform (Unipark) by researchers, following data entry rules agreed in the study consortium. For quality assurance, 10% of the paper-based survey entries will be reviewed independently by a second researcher at the study sites. After the completion of the data collection, raw data will be downloaded in analyzable formats such as IBM SPSS Statistics 25.

Concerning the formative evaluation of the e-learning, a link in the e-learning course will lead the participants directly to the corresponding online survey. Data will be collected anonymously and will be entered by participants. They were also stored at QuestBack server park. Concerning formative evaluation of the in-person training, data were collected via paper-based survey right after the in-person training. These anonymous surveys will be gathered in a mailbox. The collected data will also be entered into an online survey platform (Unipark) by researchers following the same rules as is stated above.

The problem-focused interviews will be carried out with the help of a half standardized interview guide by members of our research team. The interviewer will be trained by the consortium leaders. The interviews will be conducted via phone and recorded on tape. Before the interview, participants will sign a declaration of consent for the electronic recording, transcription and use of data for publications and lectures. In addition, they will sign an information leaflet of data confidentiality in accordance with the European General Data Protection Regulation (EU-DSGVO). The tape material will be transcripted by researchers according to transcription rules of Dresing and Pehl [64]. For the transcription and the subsequent evaluation MAXQDA Plus 12 software (VERBI Software GmbH, VERBI Software Company) will be used. The data will be stored on password-protected computers. All personal data will be deleted during the transcription process. All participants will be given a pseudonym during the evaluation to guarantee data confidentiality and ensure that no conclusions could be drawn regarding the identity of the individuals.

Due to data security aspects, data from the KOMPAS study will not be made available in the public domain. By the end of the project, survey projects will be archived and deleted from QuestBack server. All data will be stored in accordance with national and regional data security standards.

### Data analysis

Quantitative data from the pilot study will be evaluated using descriptive and inferential statistical analyses. A linear mixed model will be applied with fixed effects for the intervention group, time (repeat measures), group * time interaction, and study site, and a random intercept for ward affiliation. This model includes the estimation of the intra-cluster correlation (ICC) for our outcomes to provide the basis for a larger confirmatory study in the future. If substantial baseline differences are detected between groups, the relevant variables will be included in the analytical model at the individual or ward level. Analyses will follow the intention-to-treat approach, including data from all participants. A sensitivity analysis will be performed to assess the robustness of the findings against different strategies for dealing with missing data and accounting for the amount (“dose”) of intervention received by the participants. Potential predictors and moderators will be explored in post-hoc analyses. The qualitative data from the problem-focused individual interviews focused on reviewing the feasibility of the intervention will be analyzed on the basis of a structured contents analysis in accordance with Mayring [65–67].

In order to increase validity of the results, we will use methodological triangulation [68]. Qualitative and quantitative data will be cross-checked to search for regularities.

### Data monitoring

LK supports this study as an independent statistician. He advanced the study team in the development of the evaluation and instruments, but is not involved in trainings and the data collection process. Moreover, he is responsible for data monitoring during the data collection (e.g., cleaning the data, checking for duplicate observations), dataset preparation, and will analyze the data for primary research questions of this study. A data monitoring committee is not necessary.

## Discussion

The study puts forth a training intervention which has the potential to improve patient safety in inpatient care. Members of inpatient care teams will be trained in three key areas, which are crucial for patient safety, simultaneously. We expect the intervention to establish a standardized and quality-assured format for the training of basic competencies in Teamwork, Error management and Patient involvement. In doing so, the intervention will contribute to improving Safety-related behavior as well as Subjectively perceived patient safety by inpatient care teams. The inter-professional design of the intervention will serve to encourage exchange between the different professions in addition to establishing a shared knowledge base [69]. The blended learning format will support application of the theoretical knowledge gained, thereby facilitating the transfer into routine clinical practice.

Despite the fact that we will conduct a multicenter study, which enables a higher degree of generalization, it must be critically noted that there is a risk for selection bias in recruitment, which might narrow the external validity of the study. Possibly, hospitals and wards which choose to participate are primarily those which are prone to interventions of this type and content. Moreover, it is possible that the exclusion of pediatric, emergency, and intensive care units could narrow the generalization of the results. Furthermore, the fact that the situational judgement tests have not been validated yet, imposes a methodological limitation on the study. Future studies should establish the validity of the tools used for the specific context. Another limitation of the study are the three points of assessment. We cannot rule out that there are dropouts. That is why we assumed a high dropout rate from the beginning.

Based on former studies on interventions of patient safety [13–17], it can be assumed that the training will have great potential for the results to be used in future research and practice. The present study will provide the basis for a confirmatory phase-3 study [40]. Furthermore, the results of problem-focused individual interviews and the developed strategies for overcoming potential barriers could be used for deriving a set of implementation guidelines in healthcare. In addition, approaches for improving the e-learning course and in-person training will be developed where required in order to maximize acceptance among the participants.

In future research, the training could be offered at other hospitals. The training program could also be adapted and evaluated for the education sector and other areas of healthcare. Using a train the trainer concept, internal trainers (e.g., quality managers, human resource staff members) could be trained to perform the in-person training. Consequently, the intervention could be used continuously and comprehensively to provide advanced training for hospital staff. Moreover, it opens up the possibility of designing and specializing in a wide range of directions (e.g., tandem/team teaching, indication-specific modules).

## Trial status

Recruiting is in process (issue date 12 December 2018). Participating hospitals and wards have been provided with necessary information on intervention and data collection. Pre-data collection started in July 2018 and will probably be completed in spring 2019.

## Dissemination

The findings of the study will be communicated using a comprehensive dissemination strategy. Peer-reviewed publications are planned under corporate authorship of the KOMPAS research team. Moreover, presentations of study findings will also be taken to relevant national and international research conferences, congresses and local research symposia. Results will also be disseminated to the participating wards. Moreover, results from problem-focused interviews as well as strategies for overcoming barriers will be made publicly available to create transparency for persons in charge for the implementation of the intervention in the future.

Important protocol modifications will be reported to relevant parties (e.g., trial registries, Ethics Committee, journals) immediately.

## Additional file

Additional file 1: Standard Protocol Items: Recommendations for Interventional Trials (SPIRIT) 2013 Checklist. (DOC 126 kb)

## Data Availability

Not applicable.

## Abbreviations

BSI: Bundesamt für Sicherheit in der Informationstechnik (Federal Office for Security in Information Technology)
CIRS: Critical Incident Reporting System
CME: Continuing Medical Education
DRKS: German Register of Clinical Trials
EOQ: Error Orientation Questionnaire
EU-DSGVO: Europäische Datenschutzgrundverordnung (European General Data Protection Regulation)
FAT-K: Questionnaire on Working in a Team
G-APSQshort: German version of the Attitudes towards Patient Safety Questionnaire
HSPSC-D: Hospital Survey on Patient Safety Culture
ICC: Intra-cluster correlation coefficient
IG1: Intervention group 1
IG2: Intervention group 2
KOMPAS: German acronym for “development and evaluation of a complex training program to improve patient safety”
KSA: Knowledge, skills, abilities
MEI: Maßnahmenerfolgsinventar (activity success inventory)
NWI-R: Revised Nursing Work Index
PATEM: Patientenemfehlungen (recommendations for patients)
PPRQ: Provider-Patient-Relationship Questionnaire
SAQ: Safety Attitudes Questionnaire
SOS: Safety Organizing Scale
SUS: System Usability Scale
TeamSTEPPS: Strategies and Tools to Enhance Performance and Patient Safety
TIDieR: Templates for Intervention Description and Replication
WCG: Waiting control group
WIP: Problems in Knowledge Integration Scale

## References

[1] World Health Organization (2011). WHO patient safety curriculum guide: multi-professional edition.

[2] Vincent C (2006). Patient Safety.

[3] World Health Organization. https://www.who.int/patientsafety/en/. Accessed 10 Apr 2019.

[4] Gordon M, Darbyshire D, Baker P (2012). Non-technical skills training to enhance patient safety: a systematic review. Med Educ.

[5] Agency for Health Care Research and Quality (AHRQ). Team STEPPS. http://www.ahrq.gov/professionals/education/curriculum-tools/teamstepps/index.html. Accessed 8 June 2018.

[6] Vertino KA (2014). Evaluation of a TeamSTEPPS© initiative on staff attitudes toward teamwork. J Nurs Adm.

[7] The Joint Commission (2002). Speak up.

[8] Ginsburg L, Bain L (2017). The evaluation of a multifaceted intervention to promote “speaking up” and strengthen interprofessional teamwork climate perceptions. J Interprof Care.

[9] Okuyama A, Wagner C, Bijnen B (2014). Speaking up for patient safety by hospital-based health care professionals: a literature review. BMC Health Serv Res.

[10] Patientensicherheit Schweiz. Fehler vermeiden-Helfen Sie mit! Patientenempfehlung PATEM. https://www.patientensicherheit.ch/fileadmin/user_upload/2_Forschung_und_Entwicklung/PATEM/Web_Print_Ihre_Sicherheit_dt.pdf. Accessed 18 Aug 2016.

[11] Dinius J, Gaupp R, Becker S, Göritz AS, Körner M. Patient safety in hospitals: what we do and what we need—Focus groups with stakeholders of hospitals in Southern Germany. J Patient Saf. 2017. 10.1097/PTS.0000000000000452. 10.1097/PTS.0000000000000452 29252966

[12] Aktionsbündnis Patientensicherheit. Wege zur Patientensicherheit. 2014. http://www.pro-patientensicherheit.de/fileadmin/Medienablage/Dokumente/Aktionsb%C3%BCndnis_PatSi/APS_Lernzielkatalog_Wege_final_130206.pdf. Accessed 3 June 2019.

[13] Coburn AF, Croll Z (2011). Improving hospital patient safety through teamwork: the use of TeamSTEPPS in critical access hospitals.

[14] Morey JC, Simon R, Jay GD, Wears RL, Salisbury M, Dukes KA, Berns SD (2002). Error reduction and performance improvement in the emergency department through formal teamwork training: evaluation results of the MedTeams Project. Health Serv Res.

[15] Azimi L, Tabibi SJ, Maleki MR, Nasiripour AA, Mahmoodi M (2012). Influence of training on patient safety culture: a nurse attitude improvement perspective. Int J Hosp Res.

[16] Lisbon D, Allin D, Cleek C, Roop L, Brimacombe M, Downes C, Pingleton SK (2016). Improved knowledge, attitudes, and behaviors after implementation of TeamSTEPPS training in an academic emergency department: a pilot report. Am J Med Qual.

[17] Weaver SJ, Dy SM, Rosen MA (2014). Team-training in healthcare: a narrative synthesis of the literature. BMJ Qual Saf.

[18] Epstein NE (2014). Multidisciplinary in-hospital teams improve patient outcomes: a review. Surg Neurol Int.

[19] Buttigieg SC, West MA, Dawson JF (2011). Well-structured teams and the buffering of hospital employees from stress. Health Serv Manag Res.

[20] Becker S, Konrad A, Zimmermann L, Müller C, Tomczyk S, Reichler L, Körner M (2018). Einfluss von Teamarbeit auf Wohlbefinden und emotionale Erschöpfung von Mitarbeitern in der medizinischen Rehabilitation. Gesundheitswesen.

[21] Ross F, Elizabeth Rink, Angela Furne (2009). Integration or pragmatic coalition?: an evaluation of nursing teams in primary care. J Interprof Care.

[22] Manser T (2009). Teamwork and patient safety in dynamic domains of healthcare: a review of the literature. Acta Anaesthesiol Scand.

[23] West MA, Borrill C, Dawson J, Scully J, Carter M, Anelay S (2002). The link between the management of employees and patient mortality in acute hospitals. Int J Hum Resour Manag.

[24] Hughes KM, Benenson RS, Krichten AE, Clancy KD, Ryan JP, Hammond C (2014). A crew resource management program tailored to trauma resuscitation improves team behavior and communication. J Am Coll Surg.

[25] Brock D, Abu-Rish E, Chiu C, Hammer D, Wilson S, Vorvick L (2013). Interprofessional education in team communication: working together to improve patient safety. BMJ Qual Saf.

[26] Lee P, Allen K, Daly M (2012). A “Communication and Patient Safety” training programme for all healthcare staff: can it make a difference?. BMJ Qual Saf.

[27] Kirschbaum KA, Rask JP, Brennan M, Phelan S, Fortner SA (2012). Improved climate, culture, and communication through multidisciplinary training and instruction. Am J Obstet Gynecol.

[28] Woodward HI, Mytton OT, Lemer C, Yardley IE, Ellis BM, Rutter PD (2010). What have we learned about interventions to reduce medical errors?. Annu Rev Public Health.

[29] McEwan D, Ruissen GR, Eys MA, Zumbo BD, Beauchamp MR (2017). The effectiveness of teamwork training on teamwork behaviors and team performance: a systematic review and meta-analysis of controlled interventions. PLoS One.

[30] Omura M, Maguire J, Levett-Jones T, Stone TE (2017). The effectiveness of assertiveness communication training programs for healthcare professionals and students: a systematic review. Int J Nurs Stud.

[31] Martin JS, Ummenhofer W, Manser T, Spirig R (2010). Interprofessional collaboration among nurses and physicians: making a difference in patient outcome. Swiss Med Wkly.

[32] Hofinger G (2009). Lernen aus Fehlern im Krankenhaus. Systemische Fehlersicht und Zwischenfall-Berichtssysteme. Unfallchirurg.

[33] Schwappach DLB (2010). Review: engaging patients as vigilant partners in safety: a systematic review. Med Care Res Rev.

[34] Doherty C, Stavropoulou C (2012). Patients’ willingness and ability to participate actively in the reduction of clinical errors: a systematic literature review. Soc Sci Med.

[35] Vincent CA (2002). Patient safety. What about the patient?. Qual Saf Health Care.

[36] Härter M, Bergelt C, Mehnert A, Koch U (2016). Partizipative Entscheidungsfindung und Empowerment: Stärkung der Patientenbeteiligung in der Onkologie. Handbuch Psychoonkologie.

[37] Davis RE, Sevdalis N, Vincent CA (2011). Patient involvement in patient safety: how willing are patients to participate?. BMJ Qual Saf.

[38] Davis RE, Pinto A, Sevdalis N, Vincent C, Massey R, Darzi A (2012). Patients’ and health care professionals’ attitudes towards the PINK patient safety video. J Eval Clin Pract.

[39] Schwappach DLB, Frank O, Davis RE (2013). A vignette study to examine health care professionals’ attitudes towards patient involvement in error prevention. J Eval Clin Pract.

[40] Craig P, Dieppe P, Macintyre S, Michie S, Nazareth I, Petticrew M (2008). Developing and evaluating complex interventions: the new Medical Research Council guidance. BMJ.

[41] Chenot J (2009). Cluster-randomisierte Studien: Eine wichtige Methode in der allgemeinmedizinischen Forschung. Z Evid Fortbild Qual Gesundhwesen.

[42] Viechtbauer W, Smits L, Kotz D, Budé L, Spigt M, Serroyen J, Crutzen R (2015). A simple formula for the calculation of sample size in pilot studies. J Clin Epidemiol.

[43] Scarbath H, von Beyer-Stiepani T (2012). Handbuch Trainingskompetenz: Multiplikatorenkonzept für die betriebliche Weiterbildung.

[44] Hoffmann TC, Glasziou PP, Boutron I, Milne R, Perera R, Moher D (2014). Better reporting of interventions: template for intervention description and replication (TIDieR) checklist and guide. BMJ.

[45] Ellaway R, Masters K (2009). AMEE Guide 32. E-Learning in medical education Part 1: Learning, teaching and assessment. Med Teach.

[46] Taylor-Adams S, Vincent C (2007). Systemanalyse klinischer Zwischenfälle.

[47] Stevens MJ, Campion MA (2016). The knowledge, skill, and ability requirements for teamwork: implications for human resource management. J Manag.

[48] Christian MS, Edwards BD, Bradeley JC (2010). Situational judgement tests: constructs assessed and a meta-analysis of their criterion-related validities. Pers Psychol.

[49] Lievens F, Sackett PR, Buyse T (2009). The effects of response instructions on situational judgment test performance and validity in a high-stakes context. J Appl Psychol.

[50] McDaniel MA, Hartman NS, Whetzel DI, Grubb WL (2007). Situational judgement tests, response instructions, and validity: a meta-analysis. Pers Psychol.

[51] Körner M, Ludewig M, Becker S, Müller C, Wirtz M (2015). Kurzversion Fragebogen zur Arbeit im Team (FAT-K): internes Dokument Universität Freiburg.

[52] Warshawsky NE, Havens DS (2011). Global use of the Practice Environment Scale of the Nursing Work Index. Nurs Res.

[53] Steinheider B, Bayerl PS, Menold N, Bromme R (2009). Entwicklung und Validierung einer Skala zur Erfassung von Wissensintegrationsproblemen in interdisziplinären Projektteams (WIP). Zeitschrift für Arbeits Organisationspsychologie A&O.

[54] Rybowiak V, Garst H, Frese M, Batinic B (1999). Error orientation questionnaire (EOQ): reliability, validity, and different language equivalence. J Organ Behav.

[55] Zimmermann N, Küng K, Sereika SM, Engberg S, Sexton B, Schwendimann R (2013). Assessing the Safety Attitudes Questionnaire (SAQ), German language version in Swiss university hospitals—a validation study. BMC Health Serv Res.

[56] Kiesewetter J, Kager M, Fischer MR, Kiesewetter I (2017). Validation of a German short version of the Attitudes towards Patient Safety Questionnaire (G-APSQshort) for the measurement of undergraduate medical students’ attitudes to and needs for patient safety. GMS J Med Educ.

[57] Welp A, Meier LL, Manser T (2016). The interplay between teamwork, clinicians’ emotional exhaustion, and clinician-rated patient safety: a longitudinal study. Crit Care.

[58] Edmondson A (1999). Psychological safety and learning behavior in work teams. Adm Sci Q.

[59] Wright John, Lawton Rebecca, O’Hara Jane, Armitage Gerry, Sheard Laura, Marsh Claire, Grange Angela, McEachan Rosemary RC, Cocks Kim, Hrisos Susan, Thomson Richard, Jha Vikram, Thorp Liz, Conway Michael, Gulab Ashfaq, Walsh Peter, Watt Ian (2016). Improving patient safety through the involvement of patients: development and evaluation of novel interventions to engage patients in preventing patient safety incidents and protecting them against unintended harm. Programme Grants for Applied Research.

[60] Gremigni P, Casu G, Sommaruga M (2016). Dealing with patients in healthcare: a self-assessment tool. Patient Educ Couns.

[61] Kauffeld S, Brennecke J, Strack M, Kauffeld S (2009). Erfolge sichtbar machen: Das Maßnahmen-Erfolgs-Inventar (MEI) zur Bewertung von Trainings. Handbuch Kompetenzentwicklung.

[62] Brooke J (1996). SUS-A quick and dirty usability scale. Usability Eval Ind.

[63] Chan A-W, Tetzlaff JM, Gøtzsche PC, Altman DG, Mann H, Berlin J, Dickersin K, Hróbjartsson A, Schulz KF, Parulekar WR, Krleža-Jerić K, Laupacis A, Moher D (2013). SPIRIT 2013 explanation and elaboration: guidance for protocols of clinical trials. BMJ.

[64] Dresing T, Pehl T (2017). Praxisbuch Interview, Transkription & Analyse: Anleitungen und Regelsysteme für qualitativ Forschende.

[65] Mayring P (2015). Qualitative Inhaltsanalyse: Grundlagen und Techniken.

[66] Mayring P (2016). Einführung in die qualititative Sozialforschung: Eine Anleitung zu qualitativem Denken.

[67] Mayring P. Qualitative content analysis. Qual Soc Res. 2000. 10.17169/fqs-1.2.1089.

[68] Bekhet AK, Zauszniewski JA (2012). Methodological triangulation: an approach to understanding data. Nurse Res.

[69] Körner M, Lippenberger C, Becker S, Reichler L, Müller C, Zimmermann L (2016). Knowledge integration, teamwork and performance in health care. J Health Organ Manag.

